# The Role of Bilirubin and the Other “Yellow Players” in Neurodegenerative Diseases

**DOI:** 10.3390/antiox9090900

**Published:** 2020-09-22

**Authors:** Sri Jayanti, Libor Vítek, Claudio Tiribelli, Silvia Gazzin

**Affiliations:** 1 Fondazione Italiana Fegato-Onlus, Bldg. Q, AREA Science Park, ss14, Km 163.5, Basovizza, 34149 Trieste, Italy; sri.jayanti@fegato.it (S.J.); ctliver@fegato.it (C.T.); 2 Faculty of Medicine, Universitas Hasanuddin, Makassar 90245, Indonesia; 3 Molecular Biomedicine Ph.D. Program, University of Trieste, 34127 Trieste, Italy; 4 Institute of Medical Biochemistry and Laboratory Diagnostics, and 4th Department of Internal Medicine, Faculty General Hospital and 1st Faculty of Medicine, Charles University, 12000 Prague, Czech Republic; vitek@cesnet.cz

**Keywords:** bilirubin, bilirubin oxidation products, biliverdin, heme, heme oxygenase, biliverdin reductase, yellow players, neurodegenerative diseases, central nervous system (CNS)

## Abstract

Bilirubin is a yellow endogenous derivate of the heme catabolism. Since the 1980s, it has been recognized as one of the most potent antioxidants in nature, able to counteract 10,000× higher intracellular concentrations of H_2_O_2_. In the recent years, not only bilirubin, but also its precursor biliverdin, and the enzymes involved in their productions (namely heme oxygenase and biliverdin reductase; altogether the “yellow players”—YPs) have been recognized playing a protective role in diseases characterized by a chronic prooxidant status. Based on that, there is an ongoing effort in inducing their activity as a therapeutic option. Nevertheless, the understanding of their specific contributions to pathological conditions of the central nervous system (CNS) and their role in these diseases are limited. In this review, we will focus on the most recent evidence linking the role of the YPs specifically to neurodegenerative and neurological conditions. Both the protective, as well as potentially worsening effects of the YP’s activity will be discussed.

## 1. Introduction

**Figure 1:**
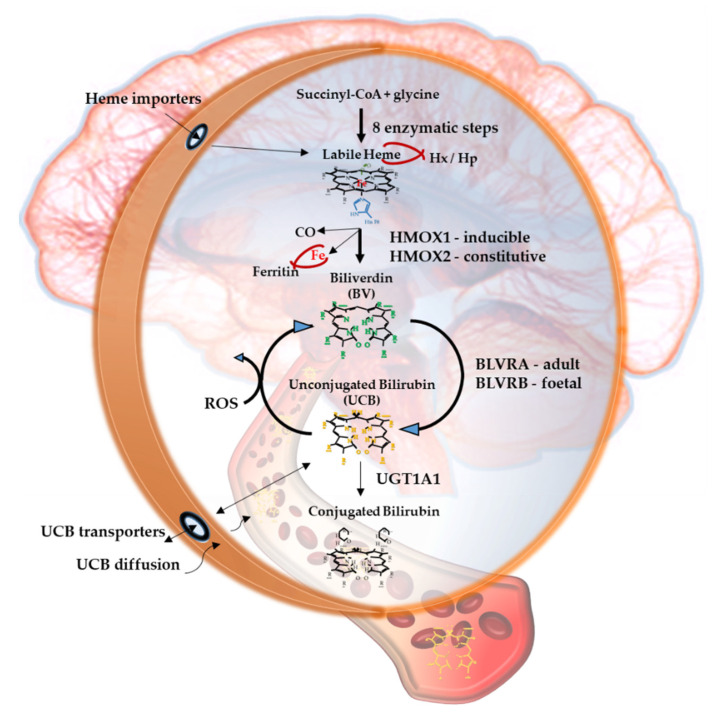
The yellow players.

Bilirubin, the end product of the consecutive enzymatic activity of heme oxygenase (HMOX) and biliverdin reductase (BLVR) (Figure 1), is mostly known as a serum marker of hepatic diseases [1,2]. Bilirubin circulates in the blood in its unconjugated form (UCB, unconjugated bilirubin) bound to albumin, with a minimal portion being unbound (free bilirubin, Bf, about 0.1% in physiological conditions) [3], and is mainly produced from heme, originating from the senescent red blood cells in the spleen. UCB is highly hydrophobic and potentially toxic in high concentrations [4,5,6], and is conjugated in the liver with 1 or 2 molecules of glucuronic acid. The formed polar conjugated bilirubin (CB), after its further metabolism in the gut lumen, is easily discarded from the body though feces. Defects in hepatic conjugation will increase the UCB content in blood, with consequent rise of the Bf fraction in serum when UCB concentration exceed the capacity of its binding compounds [3]. Due to its lipophilic properties, Bf may diffuse across the cellular bilayer entering the cells. Based on this classic concept, the blood supply has been for a longtime considered the unique source of bilirubin content in the extrahepatic tissues, including the central nervous system (CNS) [7,8].

When entering cells, UCB may counteract 10,000× higher concentrations of H_2_O_2_, being one of the most potent antioxidants in nature [3,9]. For a long time the explanation of this incredible antioxidant ability has been based on the concept of the bilirubin-biliverdin redox cycle (Figure 1), where bilirubin is oxidized back to its precursor biliverdin (BV) by reactive oxygen species (ROS), and, in turn, BV is rapidly reduced by BLVR to bilirubin [10]. As a result, the antioxidant effects of UCB is amplified without increasing the cellular concentration of the pigment to a toxic level.

Figure 1 resumes the main steps of bilirubin metabolism, as well as the basis for its antioxidant capability. The concentration of systemic (blood) bilirubin derives from the transformation of the intracellular heme (the so-called labile heme) into biliverdin (BV), together with CO and Fe^2+^, by the action of heme oxygenase (HMOX) enzymes. BV is then converted into unconjugated bilirubin (UCB) by the enzyme biliverdin reductase (BLVR). Transported to the liver by blood, UCB hydrophobic and toxic in high concentrations, is then conjugated by the uridine diphospho-glucuronosyl transferase (UGT) 1A1 to conjugated bilirubin (CB), and eliminated from the body. Inside the cell, the powerful antioxidant action of UCB is due to its conversion back to BV during the scavenging of the cellular ROS. In this BV-bilirubin redox cycle, the protection is continuously renewed maintaining the intracellular physiological concentration of the pigments. Based on this traditional concept, the main source of labile heme (thus UCB) is the turnover of the senescent red blood cells in the spleen, and the intracellular concentration of UCB in extrahepatic tissues is believed to depend on blood supply. If true, it may account for even toxic supply of heme and UCB in case of stroke or CNS conditions compromising the blood-brain interfaces. Nevertheless, recent data suggest that extrahepatic cells may produce de novo UCB, starting from a pool of labile heme that might also be replenished from both an import, as well as an in situ (intracellular) synthesis. Added to the ubiquitarian on-demand induction of HMOX and BLVR under stressor stimuli, the YPs form a local homeostatic and defensive cellular system, that might act in synergy or independently from the systemic blood bilirubin, with hemopexin (Hx), haptoglobin (Hp), and ferritin preventing the generation of ROS by the chelating/binding of free hemoglobin and iron.

Based on the recent experimental as well as clinical data not only of UCB but also of the enzymes and precursors involved in its production seem to be importantly implemented in the pathogenesis of CNS’s disorders.

Both HMOX and BLVR possess multiple binding sites for transcription factors on the promoter region of the gene, making them able to react on demand to stressor stimuli, including those characterizing the diseases [11,12,13,14,15,16], pointing to an active role in the cellular defense. In line with this concept is their induction described in several pathological conditions [1,17].

Recently, different cell types (including neuronal cells), have been demonstrated in vitro to be able to produce de novo bilirubin from its precursors, increasing cellular resistance to damage [18,19,20]. In eels, UCB cellular production and storage (UCB bind to a protein named UnaG, belonging to the fatty acid-binding protein (FABP) family) have been suggested to provide a cellular homeostatic system able to face the oxidative challenge of the eel migration [21,22]. This has not only confirmed the idea of an active role of UCB in response to stress but has underlined the importance of the cellular UCB concentration in this process.

Finally, a correlation between UCB concentration, as well as HMOX1/BLVR activation, and the diseases have been described both in the experimental and clinical studies [1,17].

Considering quite a specific environment of the CNS-highly lipophilic, with high O_2_ consumption and a limited expression of antioxidant defense, making the brain highly susceptible to oxidative stress—the modulation of bilirubin and the YPs may be an intriguing therapeutic target.

The vast majority of our current knowledge on the role of the YPs derives from extra CNS diseases (such as cardiovascular diseases, metabolic syndrome, diabetes, etc.), while what this entails specifically for the CNS is still largely unknown.

In this work, we review the key knowledge and the most recent opinions about the potential effects of the YP on the onset and progression of the neurological conditions. We highlight the association of the YP with brain diseases and address the potential molecular mechanisms involved in both protection and damage of the CNS.

## 2. The Yellow Players (YP)

### 2.1. Heme

Heme is a cyclic tetrapyrrolic molecule belonging to a superfamily of the most conserved compounds in nature [3]. Heme forms a prosthetic group for a variety of hemoproteins, the most important being hemoglobin, myoglobin and cytochromes, and is implicated in multiple cellular functions including energy generation, oxygen transport, defense against increased oxidative stress, cell signaling as well as light-harvesting in higher plants, cyanobacteria and blue-green algae [3]. As usual, heme might be toxic when surpassing certain threshold concentrations, but may also exert potent protective effects [23], and this is true also for the CNS [12,24,25,26,27] (Table 1 and Table 2). In cultured neurons, heme accumulates intracellularly and can be even more neurotoxic than iron [28]. The heme metabolism in the brain seems to be impaired in neurodegenerative diseases as documented by elevated expression of HMOX1 in these pathologies ([29], see also below). Simultaneously, hereditary defects of the heme synthesis, cellular export, and import of heme as well as impairment of its incorporation into hemoproteins or heme degradation are associated with specific neurodegenerative disorders supporting the role of heme metabolism in the brain damage [24]. The role of heme in CNS pathologies is provided by studies on intracranial bleeding demonstrating neurotoxicity of free hemoglobin and its degradation products released during hemorrhage [30].

On the other hand, heme might be neuroprotective by reducing neuronal apoptosis, improving mitochondrial functions as shown in experimental animal studies. These effects might be mediated via HMOX1 induction or by increasing the expression of neuroglobin [25,26], the hemoprotein positively correlated with a beneficial outcome in several neurotoxic insults including ischemic and traumatic brain injuries and Alzheimer’s disease [31]. Neuroprotective effects of hemin against lead neurotoxicity, also mediated by increased expression of HMOX1, were reported also in another experimental study [27].

### 2.2. Heme Oxygenase (HMOX), Carbon Monoxide (CO) and Iron

As described above, heme is involved in the pathological processes of the brain. Under physiological conditions heme homeostasis is tightly regulated by HMOX enzymes. Two HMOX isoforms exist in the human body, the inducible HMOX1, and the HMOX2 isoenzyme constitutively expressed also in the CNS [15].

In addition to converting heme to BV, HMOX1 possess a wide spectrum of DNA-binding motifs on its promoter (e.g., CRE/Erg1—cAMP response element/early growth response, NF-kB, AP2, Hif, cJun/Fos, ATF, stRE—tress response element), making it able to rapidly modulating a plethora signaling pathway involved in adaptation to stress, proliferation, differentiation and cell survival, immunity, anti-oxidant response, as well as modulating the expression of HMOX itself [1].

In the CNS context, HMOX1 is generally viewed as neuroprotective and significant effort is being made to therapeutically induce HMOX1 to prevent various neuropsychiatric and neurodegenerative diseases, either via direct HMOX1 induction or by activating its transcription factor Nrf2 by therapeutics passing the blood-brain barrier (BBB) [33,128,129]. Based on mostly experimentally studies, HMOX1 was indeed proved to protect the brain in various neurotoxicity models such as acute glutamatergic and aspartatergic excitotoxicity [35,36], ethanol-induced neurotoxicity [37], glycolysis inhibition-induced neurotoxicity and toxicity against mitochondria in cerebellar granule neurons [38,130] as well as rat model [32] (Table 1). These data supporting the protective role of HMOX1 in neurotoxicity and neurodegeneration are in line with studies by Takahashi et al. demonstrating the inhibition of HMOX in neurons of a transgenic mice model of Alzheimer’s disease [84].

On the other hand, the exaggerated activity of HMOX1 may result in an overproduction of heme-derived carbon monoxide (CO) and especially iron, leading to increased astroglial stress accompanied with oxidative mitochondrial membrane damage, iron sequestration and mitophagy, as well as to gliopathy present in many aging-related neurodegenerative brain disorders [16] (Table 1). Excessive HMOX1 overexpression was reported to contribute to the pathological iron deposition and mitochondrial damage in aging-related neurodegenerative disorders [46,131] with all the pathological consequences associated with iron accumulation in the brain tissue [45], Similarly, although CO at low doses is neuroprotective by diminishing cerebral vasospasms in subarachnoid hemorrhage [129], and by protecting neurons from toxic noxious substances [38], CO at higher concentrations is certainly toxic [47,49] (Table 1).

Not only HMOX1, but also HMOX2 constitutively expressed in the CNS is implicated in the protection from various neurological disorders as demonstrated in the experimental models of cerebral ischemia-reperfusion injury [43,132] or oxidative stress-mediated hippocampal and neuronal toxicity (Table 1).

Altogether, the current knowledge suggests HMOX, and especially HMOX2, as part of a CNS cellular defensive machinery, and (particularly the inducible HMOX1) as an interesting pharmacological target for enhancing the brain adaptation to the pathological conditions. Nevertheless, and differently for the extra-CNS organs, special care of the side effects due to an excessive HMOX1 induction, must be taken into consideration (see BLVR section and Conclusion and perspective).

### 2.3. Biliverdin

BV, the greenish, water-soluble metabolite produced by the catalytic degradation of heme by HMOX [11,133], is probably the least studied product of this enzyme. Due to its rapid reduction to UCB by BLVR [134,135], BV is almost undetectable in serum and cells [90,136,137].

**Figure 2:**
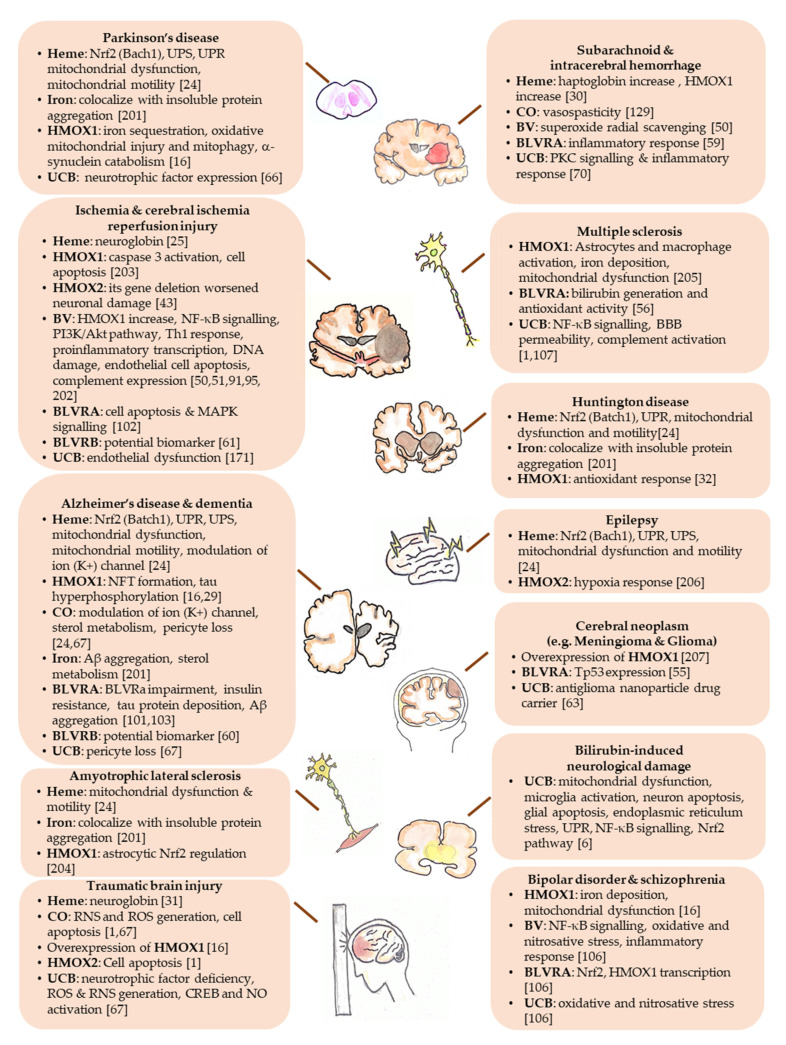
The known and the putative molecular targets of the YPs on the CNS diseases.

Nevertheless, experimental studies have demonstrated that BV administration to rats ameliorates brain damage by reducing oxidative stress, and decreasing DNA damage (Table 1 and Table 2) [50], and is a biomarker for oxidative stress in many neurodegenerative diseases (Figure 2) [138]. When administered in vivo, BV alleviates the pro-inflammatory response [51,91,92], playing a role in the progress of neurodegeneration [139], and inhibits the toll-like receptor (TLR) 4 signaling [93,96], a frequent contributor to neuronal death, BBB damage, edema, ischemic brain injury [140,141,142,143,144,145,146], and upregulated in microglia of Alzheimer’s disease patients [147,148,149]. The rapid conversion of BV to UCB still leaves open the question of which of the molecules (BV or UCB) is the more important effector.

The specific contribution of BV has been thoroughly investigated in in vitro (chemical) studies where BV has been demonstrated to scavenge NO radicals [150], and inhibit lipid peroxidation with a 2-fold higher efficacy compared to α-tocopherol [90]. This data has been supported in vivo studies with BLVRA deficient mice as well as in the cell lines in which the BLVRA was silenced [151].

Altogether, the data indicate that the protection observed both in cellular systems as well as in vivo, might be a combination of a direct antioxidant effect of BV and its conversion into bilirubin.

On the other hand, BV administration in jaundiced Gunn rats has been shown to induce abnormalities in the brainstem auditory evoked potential comparable with those observed in human newborn hyperbilirubinemia (Table 1). In this study, BV administration was followed by an increase in plasma bilirubin level, the real effector of the brain damage [52].

### 2.4. Biliverdin Reductase (BLVR)

Two isoforms of BLVR (A and B) reduce BV to UCB, and both possess kinase activity.

BLVRB is highly expressed in the early fetal stages and reduces the fetal BV IXβ, whose accumulation, together with the ferric ion derived from the heme cleavage, may leads to toxicity to the developing fetus [53,54] (Table 1). Despite detectable in the adult tissues, the role in adults has not been deciphered. Nevertheless its detection in serum has been suggested as a potential biomarker for early diagnosis of Alzheimer’s disease [60], intra-plaque hemorrhage in atherosclerosis and carotid atherosclerosis, common causes of cerebral thromboembolism or ischemic stroke [61].

BLVRA has been much more investigated. Its expression increases later in gestation [152] and is ubiquitously expressed in the adult [153], with maximal levels in the brain and lungs.

BLVR may be found both in the cytoplasm and in the nucleus. In the cytoplasm, apart from reducing BV to bilirubin IXα, it may be a substrate for the insulin receptor tyrosine kinase (IRK), and acting as a kinase on itself, as well as on several signaling pathway with important adaptive/defensive functions (e.g.,—anti-oxidant, inflammatory and hypoxia response, detoxification, apoptosis, carcinogenesis; response to insulin. For details see [1], in addition to Table 2 in this review). BLVRA may also translocate into the nucleus transporting heme and ERK (extracellular signal-regulated kinases) and act as a transcription factor binding directly to ARE (antioxidant responsive elements )/AP1-2 (activating protein), and ATF2 (activating transcription factor)/CRE (cAMP response element) DNA sequences (present also on the promoter region of HMOX1), or acting in complex with ERK/Elk (ETS domain-containing protein) or Nrf2 (nuclear factor (erythroid-derived 2)-like 2)/ARE (antioxidant responsive elements) [1,154,155,156] (Table 2). Altogether, BLVR possesses the potential for modulating a wide number of biological function in the cells, including the self-regulation of the YPs, through an impressive array of signaling pathway [1].

As a transcription factor, BLVRA binds to NF-κB, arresting the cell cycle [91]. As a consequence, BLVRA is downregulated in brain tumors, particularly meningiomas and gliomas, where a correlation between the enzyme expression and the anti-oxidant status has been found [55]. BLVRA deficiency has a role also in the maintenance of the endothelial phenotype controlled by HMOX and iron homeostasis control, with potential implications for the BBB integrity during diseases [157,158,159,160]. Deregulation of the BLVRA activity is a common feature of Alzheimer’s disease, at least in the most advanced stages, with BLVRA inhibition enhancing Tau phosphorylation and deposition in the brain [98,99,100,101] (Table 1 and Table 2). The suggested explanation for the BLVRA enzymatic inactivation lies in the excessive oxidative and nitrosative stress ongoing the disease, damaging the enzymatic functions [57,58,161], a phenomenon common in most of the neurological conditions.

Notably, BLVRA is also a member of the insulin receptor substrate family [162], modulating the glucose uptake [105,154,163] (Table 2), with insulin resistance frequently observed in Alzheimer’s disease [164,165,166]. The role of BLVRA in insulin resistance and disease progression, has been better unraveled in animals models, where the reduced BLVRA activity, the brain insulin resistance, and the disease severity, have been improved by intranasal insulin administration, the effect not occurring in the BLVRA knock-out animals [167].

Vice versa, BLVR intracranial administration in rats ameliorates the outcome of autoimmune encephalomyelitis (a model for multiple sclerosis). The efficacy has been explained by the multifactorial functions of bilirubin (anti-complement, inhibiting the antibody-dependent lymphocytes cell-mediated cytotoxicity, in addition to its antioxidant action (Table 2)).

Collectively, BLVRA induction seems always beneficial to CNS, while its enzymatic inactivation looks detrimental, possibly by reducing the final concentration of UCB inside the cell. Convincing experimental demonstrations of the role of BLVR are still required to unravel the importance of this YP per se, and the side effects linked with a hyper-activation of HMOX1.

### 2.5. Unconjugated Bilirubin (UCB)

UCB is considered a powerful anti-oxidant molecule [9,10], with its chemical characteristic contributing to the physiological implications. Bilirubin contains an extended system of conjugated double bonds and a pair reactive hydrogen atom that is involved in antioxidant activity via H-donation to an incipient radical [168]. Owing to its hydrophobic nature, bilirubin mostly accounts for the preferential scavenging of lipophilic radicals that can attack lipid membranes, with the GSH/GSSG system more active on the cytosolic protection [169].

Unlike BV that has a double bond between the inner pyrrole rings, UCB contains a single bond. This UCB electrophilic characteristic accounts for its ability to react with thiol compounds characteristic of many transcription nuclear factors [133]. Thus, UCB may modulate key signalling pathways [107,112,113] (Table 2).

Among the biological functions, UCB scavenges not only ROS [62], but also RNS (reactive nitrogen species) [90,150], with reduction of the superoxide production [114]), and inhibition of the glutamate excitotoxicity [170] (Table 2). Besides, UCB is a known multi-target anti-inflammatory molecule with the pro-inflammatory processes ever noticed in CNS diseases and co-responsible for the neurological damage [107,171] (Table 2).

These properties explain why bilirubin might play a key role in reducing neuronal damage in CNS pathologies (Table 1) [42,62,63,64,65]. Nanoparticle-delivered UCB [172] into the brain reduced the tumor size and improved the survival in a mice model of glioma [63].

An interesting correlation between the serum bilirubin and the neurological conditions is emerging. Increasing clinical observations indicate a lower serum bilirubin concentrations during oxygen radical associated and inflammatory neurological conditions of the adult life (Table 1), with both a correlation with the diagnosis and the prognosis [1,2,171]. As reviewed by Fujiwara et Al. [75], similar data are present also in neonates [72,73,74], where a close association between the plasma bilirubin concentration and the plasma antioxidant capacity has been reported [75], with icteric neonates showing a favourable plasma antioxidant capacity, that phototherapy worsened [173]. After more than a century, this supports the speculation that the production of UCB from BV, an unnecessary energy-consuming reaction, is motivated by the benefits of having higher antioxidant defense. Altogether, these data suggest that lower serum bilirubin concentrations harm the systemic antioxidant defence system, possibly starting or enhancing the progression of oxidative stress-mediated neurological diseases. The real contribution of the serum bilirubin level vs. the in situ (CNS) activity of the UCB players must be further explored.

The complexity in interpreting the interplay between the liver (as the main controller of the systemic UCB level), the brain (neurological diseases) and the YPs is also present in the non-alcoholic fatty liver disease (NAFLD), the hepatic manifestation of the metabolic syndrome. NALFD is a pandemic condition involving also the pediatric population [174,175], and regarded as one of the newest risk factors for neurological diseases [176,177], with the life style and the diet regimen being key factors in the CNS pathology progression [178,179,180]. The liver and brain appear to be inter-connected at various levels (so-called liver brain axis): (1) A negative correlation between serum bilirubin concentrations and NAFLD stage has been reported [75,181,182,183,184]; (2) the modulation of HMOX1/CO/iron, in turn acting on sirtuin1 (Sirt1—see Table 2), a histone deacetylase controlling the adaptive mechanism to disease and the bilirubin transport in both organs [89,185,186,187] has been also demonstrated; and (3), the liver and brain may be connected by insulin resistance [183], a feature of the metabolic syndrome whose CNS consequences have been discussed in Section 2.4.

On the other side, UCB in high concentrations such in severe neonatal hyperbilirubinemia may cause neurological sequelae with temporary or permanent auditory dysfunctions, cognitive and motor impairment or even death [4] due to its prooxidant, proinflammatory and proapoptotic activities as well as alteration of the epigenetic control of postnatal brain development [6,108]. At the toxic level, no doubts exist that the UCB content in the CNS is due to the pigment entering from the blood (Table 2).

### 2.6. UCB Degradation Products

Apart from the main heme catabolic pathway comprising in the reduction of double bonds within the UCB molecule and resulting in the production of a series of products known as urobilinoids [188], UCB, under conditions of increased oxidative stress or upon exposure to light, can be oxidized to several UCB oxidation products. Although these include also BV produced in the so-called bilirubin/biliverdin redox cycle scavenging the overproduction of ROS [189] (Figure 1), UCB is easily (photo)oxidized into many oxidation products with biological importance [190]. These bilirubin oxidation derivatives include tetra-, tri-, di-, and mono-pyrrolic bilirubin oxidation products. Probably most clinically important are tetrapyrrolic bilirubin photo-isomers formed during phototherapy of severe unconjugated hyperbilirubinemia. However, no solid data exists whether the bilirubin photo-isomers might have the potential to affect pathologic processes of the brain tissue. Nevertheless, bilirubin photo-isomers might have neuro-inflammatory effects, as shown in vitro [77] (Table 1). Although proinflammatory cytokines and chemokines have generally been considered deleterious for the CNS and is involved in neurodegeneration [191,192], these cytokines, apart from being mediators of damage, might also have beneficial functions, serving as trophic and/or neuroprotective agents (for review see [193]). For instance, the beneficial role of IL-6 in neuroregeneration [194], as well as increased proliferation of neural progenitor cells upon exposure to TNFα treatment [195] have been reported. More data are necessary to identify the exact roles of bilirubin photo-isomers in the biology of the cells of the CNS, compared to proved deleterious (proinflammatory) effects of high concentrations of UCB [196,197].

Biopyrrins, tripyrrolic compounds representing clinically relevant markers of increased oxidative stress, comprise another group of bilirubin oxidation products [198,199]. Although their increased urinary outputs have been reported in many human pathologies associated with increased oxidative stress, their role in brain biology or bilirubin phototherapy is unexplored and deserves further investigation. The only clinical evidence on the possible role of biopyrrins in the brain pathology is the report by Chinese researchers demonstrating increased urinary excretion of biopyrrins in patients with Parkinson’s disease [78] (Table 1).

Much more is known about dipyrrolic propentdyopents and monopyrrolic bilirubin oxidation products (*Z*-BOX A and B) which recently have been demonstrated to have potential clinical impact, especially in the pathogenesis of brain damage during subarachnoid hemorrhage [79,80] (Table 1 and Table 2). Again, further studies are desperately needed to reveal all the biological roles of these bilirubin oxidation products. The recently reported analytical method for the simultaneous determination of major bilirubin photooxidation products [200] will be instrumental.

As discussed in the text, the YPs have been demonstrated to be involved in the pathogenesis and/or in the protection in neurodegenerative diseases and other CNS diseases. This figure highlights the potential molecular targets of each one of the YPs in the specific CNS diseases, based on the available literature (see References in Figure 2), resuming and connecting the text to Table 1 and Table 2. The YPs: Aβ, amyloid β; BV, biliverdin; BVR, biliverdin reductase; CO, carbon monoxide; CREB, cAMP-responsive element-binding; HMOX, heme oxygenase; NFT, neurofibrillary tangles; Nrf2, nuclear factor erythroid 2–related factor 2; NF-κB, nuclear factor kappa-light-chain-enhancer of activated B cells; NO, nitric oxide; ROS, reactive oxygen species; RNS, reactive nitrogen species; UCB, unconjugated bilirubin; UPR, unfolded protein response; UPS, ubiquitin-proteasome system; Tp53, human p53 tumor protein.(For details see [201,202,203,204,205,206,207]).

## 3. Conclusions

Heme, UCB, BV, BVLR and HMOX, are the components of a complex cellular system. In this review, we addressed the role of each YPs on brain heath, discussing both beneficial and detrimental effects. Recent experimental and clinical studies have demonstrated their role and importance in development and progression of various neurological conditions. Future detailed and controlled studies are needed to explore precise role of all the YPs in pathogenesis of these diseases, and how to modulate the YPs in a balanced fashion to prevent or improve their course.

## Figures and Tables

**Table 1:** Association of the yellow players (YPs) with brain diseases.

Yellow Players	Pathological Condition	Ref.
Heme	Essential for oxygen storage, neurogenesis, cell survival, differentiation, circadian rhythms regulation, cellular energy production, gene and micro RNA (miRNA) processing.	[12,24]
	Accumulating in the brain in course of hemorrhage, traumatic brain injury, stroke, ischemia, and diseases with increased BBB permeability (such as Parkinson’s, Alzheimer’s, and Huntington’s disease).	[12]
	Neuroprotective against xenobiotic toxicity.	[25,26,27]
	Neuroprotective against ischemic, traumatic and neurodegenerative insults, by inducing neuroglobin.	[31]
	Protective in a pharmacological model of Huntington’s disease.	[32]
	Contributing to the progression of brain diseases (such as intracerebral/subarachnoid hemorrhage; neuropathic porphyria; Friedreich ataxia; posterior column ataxia, retinitis pigmentosa, hereditary sensory and autonomic neuropathy).	[12,24]
	Neurotoxic in brain hemorrhage.	[30]
HMOX1/2		
HMOX1	Protective in neurodegenerative and other neurological diseases (such as Alzheimer’s and Parkinson’s disease, ischemic brain injury).	[33,34]
	Protective against glutamatergic/aspartatergic excitotoxicity.	[35,36]
	Protective against ethanol-induced neurotoxicity.	[37]
	Protective against mitochondrial toxicity.	[32,38]
	Protective in a pharmacological model of Huntington’s disease.	[32]
	Reduces the progression of neuropsychiatric syndrome.	[12]
	Protective against ROS via the production of bilirubin from heme.	[3]
	Depending on specific conditions, inducing apoptosis and cell cycle arrest (thus being protective), but also capable of increasing chemoresistance and worsening the diagnosis in CNS malignancies.	[39]
	Involved in neurodegeneration (such as cerebral infarction; Alzheimer’s and Parkinson’s disease, Down syndrome, schizophrenia; stroke and CNS trauma) and brain aging when excessively expressed.	[16,40]
HMOX2	Protective from cerebral ischemia-reperfusion injury; and traumatic brain injury.	[12,41,42,43]
	Protective from oxidative stress-mediated brain injury, such as epileptic seizures	[44]
	Deletion of HMOX2 increases redox stress damage in epileptic seizures (protective).	[12]
Iron	Neurotoxic and involved in neurodegenerative diseases when accumulating in the brain (almost all neurological conditions).	[12,45,46]
CO	Neuroprotective (in low concentrations) against vasospastic reaction accompanying subarachnoid bleeding.	[46]
	Neuroprotective (in low concentrations) against toxic noxious substances.	[38]
	Neurotoxic in high concentrations.	[47]
	May impair auditory functions.	[48,49]
	May impair cognitive and olfactory functions as well as the neuroendocrine system.	[12]
Biliverdin	Protective against cerebral infarction; cerebral ischemia-reperfusion.	[50,51]
	Inducing brainstem auditory evoked potential abnormalities	[52].
	Induces fetal toxicity.	[53,54]
BLVRA/B		
BLVRA	Protective in meningioma and glioma.	[55]
	Ameliorating autoimmune encephalomyelitis (a model for multiple sclerosis).	[56]
	Involved in the pathogenesis of Alzheimer’s disease.	[29,57,58]
	Improving neurological function in germinal matrix hemorrhage (a disease of premature infants which could bring complications like developmental delay, mental retardation, hydrocephalus and cerebral palsy).	[59]
BLVRB	Potentially protective during fetal life.	[53,54]
	Biomarker for Alzheimer’s disease and ischemic stroke.	[60,61]
Bilirubin	Neuroprotective in cellular and animal models of experimental autoimmune encephalomyelitis.	[44,62,63]
	Protective in stroke and ischemia.	[64,65]
	Protective against ethanol-induced neurotoxicity.	[37]
	Protective against mitochondrial toxicity.	[38]
	Reducing tumor size and improving survival in glioma.	[63]
	Protective against neurotoxicity in Parkinson’s disease model.	[66]
	Protective in traumatic brain injury.	[67]
	Protective in asymptomatic intracranial atherosclerosis.	[68]
	Improving survival of grafted neural stem cells.	[69]
	Contributing to inflammation in intracerebral hemorrhage.	[70]
	Correlating negatively with the neuropsychiatric/neurodegenerative disorders (bipolar disorder, schizophrenia, schizoaffective disorder, Alzheimer’s disease, dementia, multiple sclerosis, cerebral infarction in adults.)	[1,2,71,72]
	Correlating with intraventricular hemorrhage, retinopathy and greater vision loss; hypoxic-ischemic encephalopathy; and neonatal encephalopathy due to hepatic injury in infants.	[72,73,74,75]
	Responsible for brain damage in severe neonatal hyperbilirubinemia (kernicterus spectrum disorder: KSD) and Crigler-Najjar type I syndrome.	[4,5,6,76]
Bilirubin degradation products		
Bilirubin photoisomers	Pro-inflammatory activities.	[77]
Biopyrrins	Increased urinary excretion in Parkinson’s disease.	[78]
Propentdyopents	Increased in cerebrospinal fluid in subarachnoid bleeding.	[79]
Z-BOX A/B	Increased in cerebrospinal fluid in subarachnoid bleeding.	[80]

Abbreviations: BBB, blood brain barrier; BLVR, biliverdin reductase; Z-BOX A/B, Z isomer of bilirubin oxidation products type.A or B; CNS, central nervous system; CO, carbon monoxide; HMOX, heme oxygenase; KSD, kernicterus spectrum disorder; ROS, reactive oxygen species.

**Table 2:** The YPs and their molecular targets.

Yellow Players	Target	Effect	Ref
Heme	Generation of ROS/RNS	Vascular hypertension and vasoconstriction. In turn, the pro-oxidant milieu increases the oxidation of hemoglobin, enhancing heme release, protein carbonylation, lipids oxidation, MMP9 release, and tissue damage.	[12,30,81]
	Activation of TLR4	Proinflammatory activity: neutrophil migration, secretion of IL8, TNFα (activating NF-κB); increased vascular permeability; edema.	[30,81]
	Nrf2/Bach1/Keap1	Inhibiting the antioxidant response.	[24]
	Activation of PI3K/Akt	Reducing apoptosis, increasing the expression of antioxidant enzymes (SOD, and HMOX1).	[26]
	Binding to hemopexin (Hx)	Chelating heme.	[12,30]
	Binding to haptoglobin (Hp)	Chelating heme via binding of hemoglobin. If not sufficient: increased CNS hemorrhage, oxidative stress, impaired brain performance, and reduced neurological activity. Marker of BBB disruption.	[12,30]
	Modulation of proteasome activity.	Impairing the activity of the ubiquitin-proteasome system; impairing the unfolded protein response (PERK/ATF6/IRE1a); and leading to the accumulation of unfolded proteins.	[24]
	Cofactor for cytochrome c and the mitochondrial electron transport chain (complexes II, III, IV)	Mitochondrial dysfunction, impairing ATP translocation into the cytoplasm; mitophagy and apoptosis. Impairing mitochondrial trafficking (especially relevant for neurons).	[24]
	Binding to Slo1 BK ion channel	Inhibiting the cellular excitability.	[12]
	Induction of neuroglobin expression	Reducing the apoptosis, cytochrome c, and mitochondrial dysfunction.	[25]
	Induction of ferritin	Chelating Fe.	[28]
	Inducing HMOX1	Increasing cell survival and reducing redox stress.	[27]
		Decreasing lipid peroxidation, increasing the expression of the anti-apoptotic Bcl2, decreasing damage.	[46]
		Possibly increasing Fe influx in mitochondria worsening the damage, increasing redox stress and inflammation.	[46]
**HMOX1/2**			
HMOX1	Production of CO, BV and Fe.	Promoting proliferation trough synthesis of cGMP (maybe acting on CREB).	[18]
		Increasing VEGF in astrocytes, leading to angiogenesis. Activating of the BDNF–TrkB–PI3K/Akt signaling with increased neuronal survival, and reduced inflammation.	[67]
		When overexpressed, increasing cholesterol synthesis and cellular efflux, with an increased presence of oxysterols (products of cholesterol oxidation). The same result is obtained by the addition of CO or iron to the culture, suggesting one or both the HMOX1 products as the real effectors.	[16,82]
		Decreasing oxidative and nitrosative stress; increasing (restored) GSH and catalase activity; reducing the release of TNFα and IL1β; reducing (restored) the GSK3 activity.	[32]
		Increasing Fe production and deposition into astroglial mitochondria, with cellular bioenergetics failure. Increasing DNA damage (8-OHdG), protein oxidation (carbonyls), and lipid peroxidation. Altering the mitochondria morphology and cellular distribution, with mitophagy and autophagy. Enhancing the conversion of catecholamines and catechol-estrogens to neurotoxic radicals, making neurons more sensitive to H_2_O_2_ and dopamine insult.	[16]
		Acutely induced after stimuli, mainly in glial cells (astrocytes, microglia). Acute up-regulation might be protective, while a chronic up-regulation may cause toxicity. Inducing Fe cell export.	[33]
		Decreasing the expression of NLRP1, possibly through the inhibition of ATF4, inhibiting the inflammasome, reducing the neuronal death by apoptosis, and improving functional recovery.	[83]
	Increasing miRNA expression (miR16, 17 and 140)	Downregulating the mitochondrial functions (including ATP production; mitochondrial antioxidant enzymes level; intrinsic apoptotic pathway; enhancement of TNFα synthesis; up-regulation of MAPK signaling to compromise he oxidative phosphorylation).	[16]
	Migrating into nuclei	HMOX1 can migrate into nuclei and act as a transcription factor of the genes involved in the cellular antioxidant response, immunity and inflammation, autophagy, hypoxia, tumor resistance, etc. However, this mechanism has not been studied in CNS diseases so far.	[1]
HMOX1/2	Amyloid protein precursor binding to HMOX1/2	Reduced HMOX1/2 activity, reduced UCB production, and increased cellular sensitivity to H_2_O_2_ and hemin toxicity.	[84]
HMOX2	Generation of CO, BV and Fe^2+^.	Fostering cell survival (via UCB action) and proliferation (via cGMP signaling).	[18]
	Basal production of UCB and CO in neurons.	CO: inducing cyclic guanylyl cyclase, in turn producing cGMP (possibly via ERK).	[33]
	Production of UCB and cGMP	Increasing neuroprotection toward redox stress.	[85]
CO	Voltage-gated K^+^ channel	Modulating the cellular excitability.	[24]
	Activation of guanylyl cyclase	Increasing cGMP, activating of cGMP protein kinase and p38 MAPK, preventing neurons degeneration, activating noradrenergic neurons, decreasing apoptosis, reducing inflammation.	[3,33,86]
	AMPK	Inhibiting AMPK activation, decreasing the toxicity of the Aβ.	[87,88]
	HIF1α	Activating the Ca channels, CAMK2B, AMPKα, increasing mitochondrial respiration.	[67]
	HMOX1	Inducing HMOX1 (through Nrf2 signaling)	[67]
	miRNA	Increasing miR-140, 17, 16. Decreasing miR-297, 206, 187, 181a, 138, 29c, in turn reducing the mRNA levels of Ngfr, Vglut1, MAPK3, TNFα, and Sirt1, abnormally expressed in various central nervous system disorders.	[89]
Iron	Generation of hydroxyl radicals.	Inducing a high rate of protein carbonylation, reducing SOD activity, increasing DNA damage, inhibiting the DNA repair system. Activating NF-κB and AP-1 with BBB disruption and worsening of damage. Reducing mitochondrial respiratory functions.	[81]
	Activation of NF-κB and induction of inflammation.	Inducting the glutamate excitotoxicity (release of glutamate) with increased BBB permeability, neuronal autophagy, neuronal atrophy and death. Releasing of MMP9, TNFα, IL1β, microgliosis.	[81]
	miRNA	Increasing miR-140, 17, 16. Decreasing miR-297, 206, 187, 181a, 138, 29c, in turn reducing the mRNA levels of Ngfr, Vglut1, MAPK3, TNFα, and Sirt1, abnormally expressed in various CNS disorders.	[89]
BV	Scavenging ROS	Lowering DNA damage (8-OHdG).	[51,90]
	miR-204-5p, Ets1	Lowering Th1 type response.	[51]
	NF-κB	Lowering NF-κB-DNA binding and pro-inflammatory factors transcription/production	[91,92]
	TLR4	Inducing BLVR translocation into the nucleus, binding to TLR4 promoter, repressing the expression of TLR4, and leading to the inhibition of inflammatory cytokine production.	[93]
	JNK	Reducing the JNK activation, affecting JNK/AP-1 pathway, suppressing the transcription of TNFα and diminishing endothelial cell apoptosis.	[94]
	PI3K/Akt	Inducing the interaction of BLVRA with PI3K, activating Akt signaling, and increasing anti-inflammatory cytokine (IL10) production in macrophages.	[95]
	ROS, NRS formation	Preventing oxidative damage in rat brain microsomes	[50,90]
	Complement	Inhibiting C5aR gene and protein expression that is mediated by mTOR pathway accompanied by the reduction of pro-inflammatory cytokines (TNFα and IL6) gene expression (macrophages).	[93]
	BLVR	Inducing BLVR translocation into the nucleus.	[96]
	Histones	Possible inhibition of the histone synthesis.	[97]
BLVRA	Akt gene	Modulating glycogen synthase kinase, and Tau protein deposition in the brain.	[98,99,100,101]
	NF-κB	Direct binding, arresting the cell cycle.	[91]
	eNOS/NO/TLR4 pathway	Binding on the gene promoter, inhibition of transcription, reducing the inflammation.	[96]
		Improving hematoma resolution and neurological functions.	[59]
	MAPK/PI3K	Maintaining the synaptic plasticity, memory consolidation, inducing the genes required for neuronal and synapse growth, maintenance and repair processes.	[29]
	MAPK/Akt	Inhibiting MAPK/Akt activation, reducing apoptosis, protecting the hippocampal neuronal cell from oxidative stress.	[102]
	BACE-1 protein	Reducing the BLVRA activation inducing the phosphorylation of BACE-1, promoting insulin resistance and increasing Aβ levels in the brain of an animal model of aging.	[103]
	MEK1-ERK1/2-Elk1 signaling	Transcriptional activation of stress-induced genes, including HMOX1.	[104]
	Insulin receptor	Inducing an early activation of IRS1 and improving brain insulin resistance.	[105]
	HMOX1	Improving cellular antioxidant defense via HMOX1 induction.	[106]
UCB	NF-κB	Direct binding, arresting the cell cycle.	[91]
		Preventing NF-κB-DNA binding, suppressing T-cell activation.	[107]
	Histone acetylation	Modulating histone 3 acetylation and the transcription of genes involved in brain development.	[108]
	ER	Inducing ER stress, inflammation and apoptosis	[109]
	Nrf2	Activating the Nrf2 pathway, thus the antioxidant response.	[110]
	CREB	Increasing the phosphorylation of CREB possibly leading to BDNF production, boosting the survival and the repair processes in traumatic brain injury.	[67]
	AKT	Enhancing blood flow and regeneration in ischemic injury.	[67]
	HIF1α	Stabilizing HIF1α, activating Ca channels, CAMKβ, AMPKα, and increasing mitochondrial respiration.	[67]
	AhR	Modulating the transcription of genes coding for detoxification enzymes (CYP1A1, UGT1A1), acting on the cell cycle, MAPK cascade, Nrf2 pathway, and immune response.	[111,112]
	CAR	Involved in the disposal of exogenous and endogenous substances, and inhibition of gluconeogenesis.	[113]
	ApoD	A non-albumin carrier of bilirubin in human plasma, contributing to protection against oxidative stress, is highly expressed in the brain.	[113]
	Neurotrophic factor	Increasing the expression of BDNF in neurons and GDNF in glia, leading to reduction of neuronal loss in substantia nigra in animal model of Parkinson’s disease	[66]
	MRGPRX4	Mediating the cholestatic itch in the primary sensory neurons, acting in host defense and immune reaction.	[113]
	Angiogenic and energy-sensing genes in astrocytes	Enhancing PGC1α and HIF1α production in astrocytes which plays a role in mitochondria biogenesis, reduction of inflammatory, and angiogenesis	[67]
	ROS/RNS generation	Inhibiting of NMDA excitotoxicity, preventing neuronal death.	[104,114]
		Affecting BBB permeability and preventing inflammatory cell invasion	[62]
	Macrophages and T cells	Immuno-modulatory activity by reducing the expression of Fc receptor in macrophage and inhibiting T cell response.	[115]
	PKC/ICAM-1 signaling,	Contributing to neutrophil infiltration, early inflammation, and edema. Decreasing nitrate/nitrite formation, reduced perihematomal microgliosis.	[70]
	Mrp1/ABCc1	Upregulating and translocating its transporter (Mrp1/ABCc1) from the Golgi apparatus to the plasma membrane.	[116]
	Cellular and subcellular membranes	Altering membrane polarity and fluidity, the opening of the permeability transition pores, inducing cellular energy failure, activating the mitochondrial apoptotic pathway.	[117,118]
	P38MAPK-JNK1/2-NFkb	Inducting of inflammation, the release of TNFα, IL1β, reduction of the cellular viability	[119,120]
	NMDA receptors, glutamate	Inducing glutamate excitotoxicity, reducing the expression of the NMDA receptors, impairing long-term potentiation and long-term depression	[121,122,123]
	ERK-Akt-CREB	Scavenging ROS, decreasing neurotrophic factor availability.	[124]
	Mrp1/ABCc1 and Pgp/MDR1/ABCb1	Modulating the expression of its transporters (Mrp1/ABCc1 and Pgp/MDR1/ABCb1) at the blood-brain interfaces	[125]
	DNA	Inducing ROS, which in turn leads to DNA damage, despite the activation of the DNA repair pathways.	[126]
	Cell cycle	Inducing cell cycle arrest	[127]
UCB degradation products	Ca channels	Opening of the Ca channels and decreasing the conductance of the cerebral myocytes, inducing vasoconstriction.	[79]

The present table is not intended to collate all the known targets and molecular mechanisms modulated by the YP, but it is restricted solely to the direct evidence of their effects in CNS biology. Abbreviations: Aβ, amyloid β; Akt, protein kinase B; ATF6, activating transcription factor 6; ATF4, activating transcription factor 4; ATP, adenosine triphosphate; AMPK, 5′ adenosine monophosphate activated protein kinase; AP-1, activator protein 1; AMPKα, 5′ AMP-activated protein kinase alpha; AhR, aryl receptor; ApoD, apolipoprotein D; Bcl2, B-cell lymphoma 2; BACE-1, β-site APP cleaving enzyme 1; Bach1, the transcription factor BTB and CNC homology 1;.BBB: blood brain barrier; BDNF, brain-derived neurotrophic factor; BLVR, biliverdin reductase; BV: biliverdin; C5aR, complement receptor 5a; CAMKβ, Ca-calmodulin-dependent protein kinases beta; CAMK2B, Ca-Calmodulin dependent protein kinase β; CAR, constitutive androstane receptor; cGMP, cyclic guanosine monophosphate; CNS, central nervous system; CO: carbon monoxide, CREB, cAMP responsive element binding; CYP, cytochromes P450; Elk1, ETS Like-1 protein; eNOS, endothelial nitric oxide synthase; ER stress, endoplasmic reticulum stress; ERK, extracellular signal-regulated kinases; GSH, glutathione; GSK3, glycogen synthase kinase 3β; GDNF, glial cell line-derived neurotrophic factor; H_2_O_2_, hydrogen peroxide; HIF1α, hypoxia-inducible factors; HMOX1, heme oxygenase 1 Hp, haptoglobin; Hx, hemopexin; IRS1, insulin receptor substrate-1; IRE1a, serine/threonine-protein kinase/endoribonuclease inositol-requiring enzyme 1 α; IL: interleukin; MMP9: metalloproteinase 9; ICAM-1, intracellular adhesion molecule 1; JNK, c-Jun NH2-terminal kinase; Keap, Kelch-like ECH-associated protein 1; miRNA, micro RNA; MAPK, mitogen-activated protein kinase; MEK1, mitogen-activated protein kinase kinase; MRGPRX4, Mas-related G protein-coupled receptor X4; mTOR, mammalian target of rapamycin; Mrp1/ABCc1, multidrug resistance protein 1/ATP binding cassette protein c1; Ngfr, neuronal growth factor receptor; NLRP1, nod-like receptor protein 1; NO, nitric oxide; NF-κB, nuclear factor kappa-light-chain-enhancer of activated B cells; Nrf2, nuclear factor erythroid 2-related factor; NMDA, N-methyl-d-aspartic acid; PI3K: phosphatidylinositol 3-kinase; PERK: protein kinase RNA-like endoplasmic reticulum kinase; PGC-1α: peroxisome proliferators-activated receptor γ (PPARγ)-coactivator-1α; Pgp/MDR1/ABCb1, P-glycoprotein/multidrug resistance protein1/ATB binding cassette protein b1; PKC, protein kinase C; RNS, reactive nitrogen species; ROS: reactive oxygen species; Sirt1, Sirtun 1; Slo1 BK, Ca2+- and voltage-activated K+ channel; SOD, super oxide dismutase; TLR4, toll like receptor 4; TNFα, tumor necrosis factor alpha; TrkB, tropomyosin receptor kinase B; Th1, T helper cells; UCB, unconjugated bilirubin; UGT, uridine 5′-diphospho-glucuronosyltransferase; VEGF, vascular epithelial growth factor; Vglut1, vesicular glutamate transporters, 8-OHdG, 8-hydroxy-2′ deoxyguanosine (marker of DNA damage).
